# Bifurcation-based adiabatic quantum computation with a nonlinear oscillator network

**DOI:** 10.1038/srep21686

**Published:** 2016-02-22

**Authors:** Hayato Goto

**Affiliations:** 1 Frontier Research Laboratory, Corporate Research & Development Center, Toshiba Corporation, 1, Komukai Toshiba-cho, Saiwai-ku, Kawasaki-shi, 212-8582, Japan

## Abstract

The dynamics of nonlinear systems qualitatively change depending on their parameters, which is called bifurcation. A quantum-mechanical nonlinear oscillator can yield a quantum superposition of two oscillation states, known as a Schrödinger cat state, via quantum adiabatic evolution through its bifurcation point. Here we propose a quantum computer comprising such quantum nonlinear oscillators, instead of quantum bits, to solve hard combinatorial optimization problems. The nonlinear oscillator network finds optimal solutions via quantum adiabatic evolution, where nonlinear terms are increased slowly, in contrast to conventional adiabatic quantum computation or quantum annealing, where quantum fluctuation terms are decreased slowly. As a result of numerical simulations, it is concluded that quantum superposition and quantum fluctuation work effectively to find optimal solutions. It is also notable that the present computer is analogous to neural computers, which are also networks of nonlinear components. Thus, the present scheme will open new possibilities for quantum computation, nonlinear science, and artificial intelligence.

Nonlinearity is the origin of various interesting phenomena, such as chaos, fractal, and bifurcation^1^, where a bifurcation is a parameter-dependent qualitative change in nonlinear dynamics, such as a transition from a single stable state to two stable ones (bistability). As a result of recent advances in nanotechnology, artificial nonlinear oscillators may possess both large nonlinearity and low loss simultaneously and consequently enter the quantum regime^2^. A remarkable example is the generation of a quantum superposition of two oscillation states, known as a Schrödinger cat state^3^. Although a scheme for quantum computation using such cat states as quantum bits (qubits) has been proposed^4^, continuous degrees of freedom of quantum nonlinear oscillators have not been fully harnessed.

Here we first show that a quantum-mechanical nondissipative oscillator with desirable nonlinearity can yield a cat state via quantum adiabatic evolution through its bifurcation point. Next, we propose a quantum computer comprising such quantum nonlinear oscillators, which exploits a superposition of an exponentially large number of states of the nonlinear oscillator network to solve hard combinatorial optimization problems. The network finds optimal solutions via quantum adiabatic evolution. In contrast to conventional adiabatic quantum computation^5^,^6^ or quantum annealing^7^,^8^,^9^, where quantum fluctuation terms are decreased slowly, in the present computation nonlinear terms are increased slowly. To distinguish them, we refer to the present approach as bifurcation-based adiabatic quantum computation. Finally, we present numerical simulation results indicating that quantum superposition and quantum fluctuation work effectively to find optimal solutions.

## Results

### Generation of a Schrödinger cat state with a Kerr parametric oscillator

Recently, deterministic cat-state generation has been demonstrated in two different ways with superconducting microwave resonators coupled to a superconducting artificial atom^3^,^10^. Here we present a new method for deterministic cat-state generation using a nonlinear oscillator described below. This generation is based on quantum adiabatic evolution through its bifurcation point. The nonlinear oscillator is the fundamental component of our quantum computer.

The oscillator used here is a parametrically driven Kerr (or Duffing) nonlinear oscillator^2^ (Kerr parametric oscillator or KPO for short). Interestingly, this is similar to a swing, where a pendulum (approximate Duffing oscillator) is driven by modulating the eigenfrequency by changing the height of the center of mass (parametric driving). This model is not only the simplest one for the present purpose but also physically feasible. Promising candidates for implementing this model are superconducting microwave resonators with Josephson junctions^3^,^10^,^11^,^12^,^13^, nanoelectromechanical systems^14^,^15^, and carbon nanotubes^16^ (also see ref. 2).

In a frame rotating at half the pump frequency of the parametric drive and in the rotating-wave approximation, its Hamiltonian is given by^2^


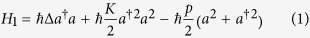


where *a* and *a*^†^ are the annihilation and creation operators for quanta of the oscillator (the quanta are, e.g., photons for electromagnetic resonators or phonons for mechanical oscillators), Δ is the detuning of the oscillator eigenfrequency from half the pump frequency, *K* is the Kerr coefficient for the Kerr effect, and *p* is the pump amplitude for the parametric drive. Hereafter, we assume that *K* and Δ are positive constants and *p* is a nonnegative control parameter. When *K* is negative, similar discussion is straightforward.

A cat state is generated via quantum adiabatic evolution as follows. Initially, the state of the oscillator and the pump amplitude are set to the “vacuum” 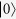 and zero, respectively. Note that 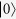 is the ground state of *H*_1_ with *p* = 0. As *p* is increased slowly, the system follows adiabatically the ground state of *H*_1_(*t*). When *p* becomes much larger than Δ, the final state becomes approximately the ground state of *H*_1_ with Δ = 0, which is a superposition of two coherent states 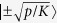. (A coherent state 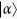 is defined as the eigenstate of *a*: 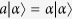
17.) Since *H*_1_ is symmetric under parity inversion 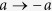, the final state should have the same parity as 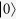. Consequently, the final state becomes approximately the cat state 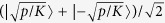, where 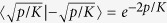 has been ignored assuming sufficiently large *p*.

**Figure 1:**
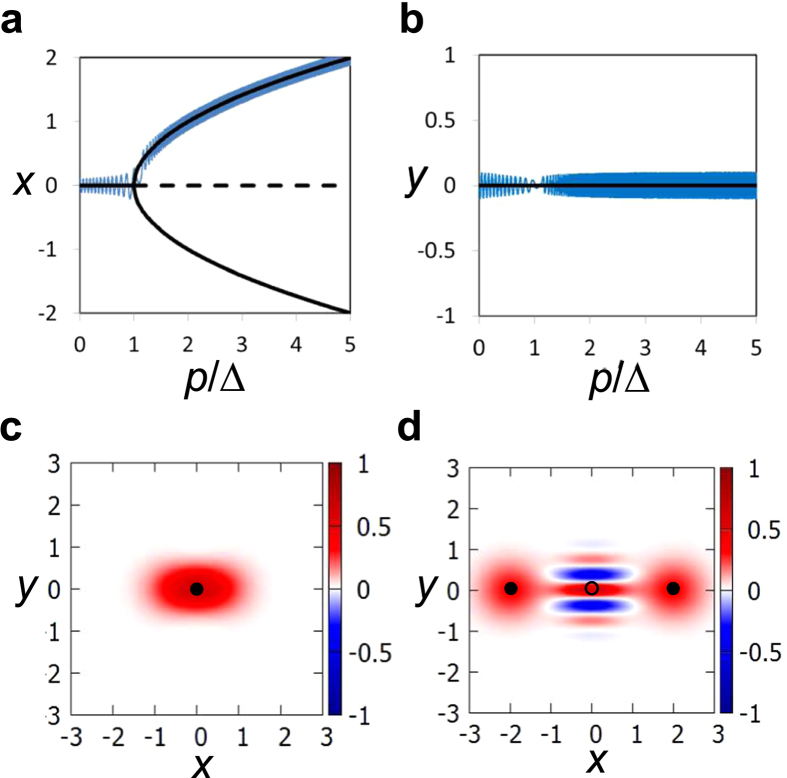
Dynamics of a single KPO. Here Δ is set to *K*. (**a**,**b**) Bifurcation diagrams (bold lines) and the simulation results (thin lines) for the classical model. The solid and broken bold lines correspond to the stable and unstable fixed points, respectively. (**c**,**d**) The Wigner functions obtained by numerically solving the Schrödinger equation with *H*_1_ in Eq. (1). 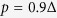 in **(c)** and 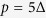 in (**d**). In (**c**,**d**), the filled and open circles represent the stable and unstable fixed points, respectively, in the classical model.

This result can be understood qualitatively from a viewpoint of classical nonlinear dynamics as follows. A classical model for the above system is defined by


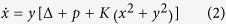



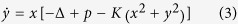


where *x* and *y* are real variables corresponding to the Hermitian operators 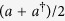 and 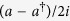, respectively, and the dots denote differentiation with respect to time *t*. These equations are derived by replacing *a* with *x *+ *iy* in the Heisenberg equations of motion for *a* with *H*_1_. This model exhibits a bifurcation at *p* = Δ as follows. When *p* ≤ Δ, the origin is a single fixed point, which is stable. (Fixed points are defined by 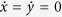
^1^.) When *p *> Δ, the origin becomes an unstable fixed point and two stable ones are created, the positions of which are 
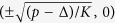
. The dependence of the fixed points on *p* is depicted in Fig. 1a,b as the bold lines, where the solid and broken lines correspond to the stable and unstable fixed points, respectively. (In Fig. 1Δ is set to *K*.) Such figures are called bifurcation diagrams^1^. The oscillating thin curves in Fig. 1a,b are obtained by numerically solving Eqs (2) and (3), where *p*(*t*) is increased linearly from *p*(0) = 0 to 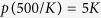 and the initial condition is set as 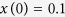 and 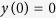. It is found that the state changes along one of the stable branches. This result is explained by the adiabatic theorem in classical mechanics^18^ (see Supplementary Information for details). Thus, the cat-state generation described above is interpreted as a superposition of two coherent states corresponding to the two classical stable branches. In other words, while the classical system chooses one of the two branches (which branch the system will choose may be unpredictable because of chaos), the quantum system can follow both the branches “simultaneously” as a superposition state. To emphasize this nonclassical feature of a quantum nonlinear oscillator, we refer to such an intriguing process as a quantum-mechanical bifurcation.

We performed numerical simulation of the cat-state generation. In this simulation, the Schrödinger equation with *H*_1_ is numerically solved, where the Hilbert space is truncated at a “photon” number of 20, the initial state is set to 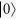, and *p* is increased linearly from 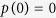 to 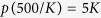. Figure 1c,d show the Wigner function 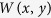 at 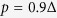 and 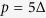, respectively. Here the Wigner function is a quasiprobability distribution for *x* and *y*^3^,^10^,^17^ (also see Methods). In Fig. 1c,d, filled and open circles represent classical stable and unstable fixed points, respectively. Note that the peaks of the Wigner functions are at the classical stable fixed points. The Wigner distributions show that the stable fixed points are accompanied by quantum fluctuations due to the Heisenberg uncertainty relation for 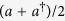 and 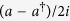. On the other hand, the interference fringe between the two peaks in Fig. 1d means that the two oscillation states are superposed^17^, that is, a cat state is generated successfully. The negative values of 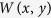 in Fig. 1d indicate the nonclassicality of the cat state.

To realize the cat-state generation, we require a large Kerr coefficient compared to a loss rate. While this requirement is too stringent for optical and mechanical systems, superconducting circuits with Josephson junctions have already achieved this situation^11^,^12^. Thus, superconducting systems are the most promising for implementing the present scheme.

### Adiabatic quantum computation with a KPO network

Next, our quantum computer with KPOs is described. If we have *N* independent KPOs, we will obtain a superposition of 2^*N*^ oscillation states via the quantum-mechanical bifurcation described above. To exploit the exponentially large number of states for solving combinatorial optimization problems, we couple the KPOs to one another appropriately depending on given problems.

The combinatorial optimization problem studied here is the Ising problem: given a dimensionless Ising energy


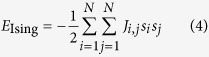


we want to find a spin configuration minimizing *E*_Ising_. Here the Ising spin *s*_*i*_ takes 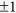, *N* is the number of spins, and the coupling coefficients satisfy 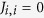 and 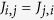. Here note that two configurations 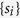 and 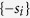 give the same Ising energy, and therefore there are always two solutions for each problem. This Ising problem is extremely hard unless the coupling topology is too simple; more precisely, it is known to be non-deterministic polynomial-time hard (NP-hard) in computational complexity theory^19^. Recently, machines specially designed for the Ising problem have attracted much attention^20^,^21^,^22^,^23^,^24^,^25^,^26^,^27^.

For the above problem, we couple *N* KPOs as follows:


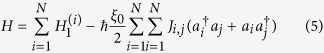


where 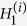 is the Hamiltonian for the *i*th KPO of the form of Eq. (1) with an individually controllable detuning Δ_*i*_ and 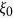 is a positive constant with the dimension of frequency. Note that the coupling Hamiltonian describes standard linear couplings, and therefore is physically feasible. It is also notable that *H* is symmetric under *simultaneous* parity inversion defined as 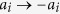 for all *i* simultaneously. In the following, we show that the KPO network can solve the Ising problem via quantum adiabatic evolution.

To use a quantum adiabatic evolution for finding a configuration minimizing *E*_Ising_, the initial state 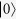 should be the ground state of *H* with *p* = 0. This condition can be satisfied by setting the detunings such that the following matrix *M* becomes positive semidefinite (see Supplementary Information for the proof):


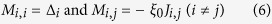


A simple setting satisfying this condition is as follows (see Supplementary Information):


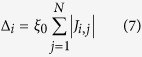


By increasing *p* slowly, we obtain the ground state of *H* with large *p* assuming that the so-called adiabatic condition^5^,^9^,^28^ is satisfied.

When *p* becomes much larger than 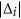 and 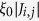, the nonlinear terms in *H* are dominant, the ground state of which is 2^*N*^-fold degenerate and the eigenspace is spanned by the tensor products of 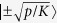. By the perturbation theory to the lowest order^28^, the correction to the energy of a tensor product 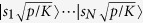 (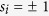) is given by


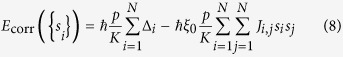


where 
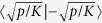
 has been ignored assuming sufficiently large *p*. Note that the first term in the right-hand side of Eq. (8) is independent of 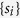 and the second one is proportional to the Ising energy. Consequently, the ground state is two-fold degenerate and the eigenspace is spanned by 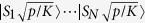 and 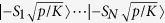, where 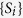 and 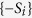 are the two solutions of the Ising problem. The degeneracy comes from the simultaneous parity symmetry of *H*. Taking the simultaneous parity symmetry of *H* into account, the ground state obtained as a final state in the above adiabatic evolution is given by


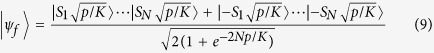


Thus, it turns out that we can find a solution of the Ising problem by measuring the amplitudes of the KPOs and identifying their signs with the Ising spins.

The above degeneracy means that the energy gap between the ground state and the first excited state will vanish, which seems problematic for the adiabatic approach. However, this causes no problem for the following two reasons. First, the transition between the two states is prohibited by the simultaneous parity symmetry of *H*. (The ground and first excited states have even and odd parities, respectively.) Second, even if the transition occurs by some accidental errors, we can find a solution correctly because the first excited state is also a superposition of 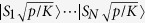 and 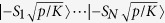 (the same as 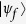 except for a negative relative phase).

In the above discussion, we have shown that the KPO network can solve the Ising problem by assuming the adiabatic condition. From the adiabatic condition, it turns out that the speed of adiabatic quantum computation is limited by the minimum energy gap between the ground and excited states^5^,^9^. Thus, the study of the energy level structure is important for evaluating and improving the computational speed^29^,^30^,^31^. In the present scheme, however, the energy level structure may be much more complex than the ones in conventional adiabatic quantum computation, because the fundamental component of our quantum computer is a nonlinear oscillator described by an infinite-dimensional Hilbert space, not a simple two-level system (qubit). In the present work, we instead performed numerical simulations, the results of which support the above discussion (see below).

It is also notable that an entangled cat state given by Eq. (9) is generated as a result of the quantum computation. We confirmed this fact by numerical simulation in the case of two spins (see Supplementary Information). Thus, the present scheme also provides a method for the generation of the intriguing states via quantum adiabatic evolution.

### Numerical simulation results

Finally, we present numerical simulation results of the quantum computation for four-spin problems, which are more difficult than two- and three-spin ones in the sense that, in the four-spin case, there may be not only frustration but also a nonglobal local minimum. In these simulations, the Schrödinger equation with *H* in Eq. (5) is numerically solved, where the Hilbert space is truncated at a “photon” number of 18 for each KPO, the initial state is set to 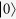, 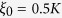, the detunings are set as Eq. (7), and *p* is increased linearly from 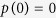 to 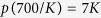.

**Figure 2:**
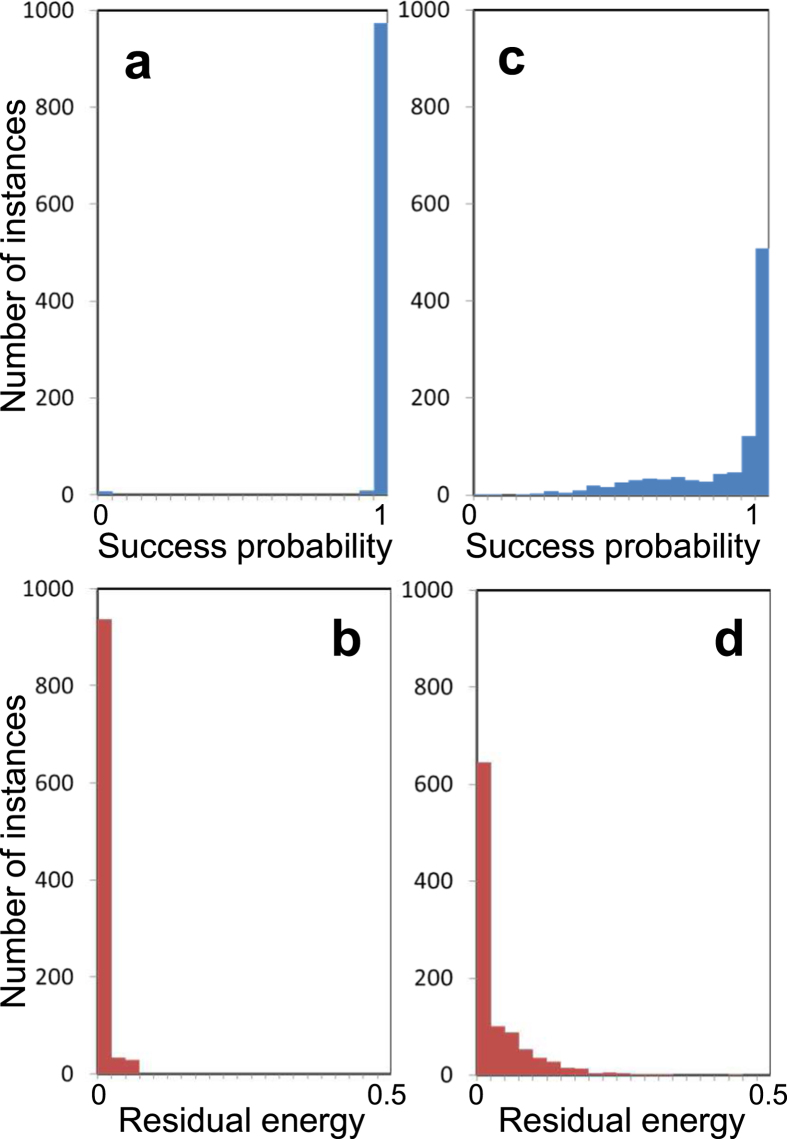
Simulation results for four-spin Ising problems. (**a**,**b**) Histograms for the success probabilities and the residual energies, respectively, in the quantum computation, estimated by numerically solving the Schrödinger equation with *H* in Eq. (5). (**c**,**d**) Histograms for the success probabilities and the residual energies, respectively, for the classical model.

We generated 1000 instances of the problem with the coupling coefficients chosen randomly in the range 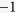 to 1. We estimated the success probability and the residual energy for each instance (see Methods for details), where the residual energy is defined as the difference between the Ising energy obtained by simulation and its minimum value^8^. The histograms of the success probabilities and the residual energies are shown in Fig. 2a,b, respectively. In Fig. 2, we treat two configurations 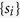 and 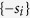 as a pair because these give the same value of *E*_Ising_.

We also simulated a classical model for the quantum computation, the equations of which are derived in a similar manner to Eqs (2) and (3) (see Supplementary Information for details). From the results for a single KPO, the comparison between the quantum and classical models may be helpful for understanding the simulation results. For each instance, we repeated the classical simulation 10^3^ times, setting the initial values of *x*_*i*_ and *y*_*i*_ to random numbers in the range −10^−6^ to 10^−6^. The success probability and the residual energy for each instance are estimated by taking averages. The results are shown in Fig. 2c,d.

## Discussion

Here we discuss the results shown in Fig. 2.

First, it is notable that the classical model can find optimal solutions with high probability. This result comes from the fact that the classical model can approximately solve the Ising problem (see Supplementary Information). The high success probability for the classical model means that the approximation is fairly good. (Thus, this model may provide a new approach to combinatorial optimization problems with nonlinear dynamics. Although the study of the classical model, which may exhibit chaotic behaviors, is interesting, this is beyond the scope of the present work.)

Next, it is clear that the quantum model can achieve higher performance than the classical one. Since the differences between the two models may be mainly quantum superposition and quantum fluctuation, the high performance may come from these quantum effects. To examine this point, we look into one of the most difficult instances, for which the classical model almost always fails.

**Figure 3:**
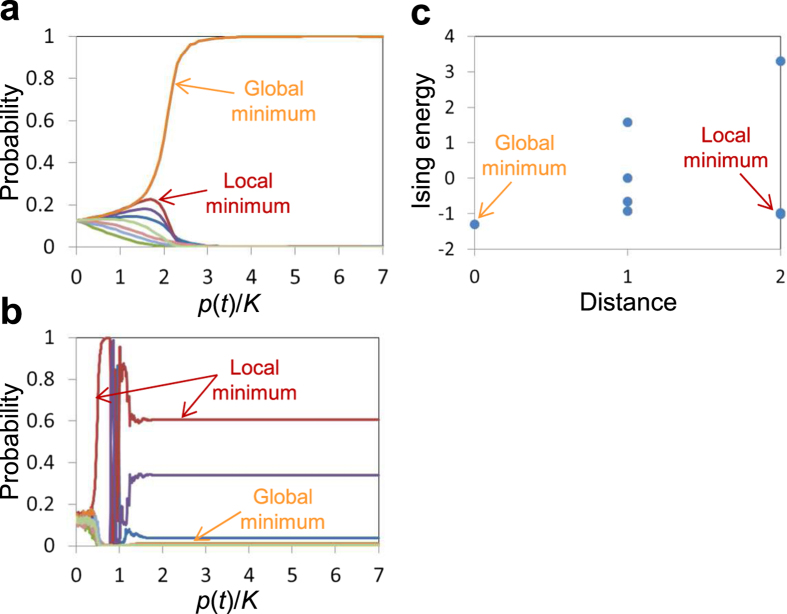
Simulation results for a difficult problem. (**a**,**b**) Time evolutions of the probabilities for the spin configurations in the quantum (**a**) and classical (**b**) models for one of the most difficult instances, for which the classical model almost always fails. **(c)**, Energy landscape of this instance, where the distance is defined by Eq. (10). Here two configurations 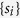 and 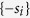 are treated as a pair because these give the same value of *E*_Ising_.

The time evolutions of the probabilities for the spin configurations in the quantum and classical models are shown in Fig. 3a,b, respectively. Figure 3c shows the energy landscape of this instance, where the distance between a configuration 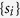 and the solution 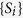 is defined as


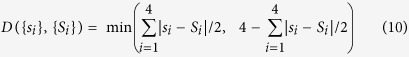


In this problem, there are two local minima. The classical model is trapped around the nonglobal local minimum (Fig. 3b). On the other hand, in the quantum model, a superposition of the two local minima arises from quantum fluctuations, and finally the probability for the global minimum converges to unity via quantum adiabatic evolution (Fig. 3a). From this result, we conclude that quantum superposition and quantum fluctuation work effectively to find optimal solutions in the present computation. (Although a more detailed comparison between the two models is interesting, which may become a new theme in the field of quantum chaos, this is beyond the scope of the present work.)

While most previous computational models for quantum computation use qubits as fundamental components^5^,^32^,^33^,^34^, the present model uses quantum nonlinear oscillators. Moreover, this harnesses continuous degrees of freedom of a nonlinear oscillator network, that is, its quantum-mechanical bifurcation based on quantum adiabatic evolution for quantum computation for the first time. It is also notable that the present model is analogous to a neural network^35^. Thus, the present scheme will open a new paradigm in the fields of quantum computation, nonlinear science, and artificial intelligence.

## Methods

### The Wigner function

The Wigner function 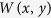 is a quasiprobability distribution for *x* and *y* corresponding to the Hermitian operators 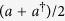 and 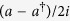, respectively, defined in the case of a single oscillator as follows^17^:


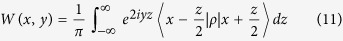


where ρ is the density operator for the system and 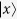 denotes the eigenstate for 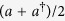. The Wigner function has a useful property that the integration with respect to *y* from 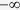 to 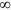 gives the probability distribution for *x*, and vice versa. Although we focus on the case of a single oscillator in the following, the generalization to multiple oscillators is straightforward. Using the displacement operator 
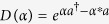
 and the parity operator 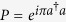, the Wigner function is rewritten as follows^17^:


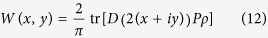


From this formula, the Wigner function in the number representation is given by


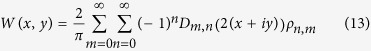


where *ρ*_*n,m*_ and 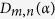 are matrix elements of *ρ* and 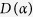, respectively, in the number representation. 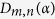 can be expressed as


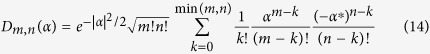


We used Eqs (13) and (14) for Fig. 1c,d.

### Calculations of success probabilities and residual energies

The success probability in Fig. 2a is defined as the probability that the spin configurations corresponding to the solution are obtained in the final measurement. Here we explain how to calculate the probability that each spin configuration is obtained. The residual energies in Fig. 2b are obtained by taking expectation values with the probabilities. Although we focus on the case of a single oscillator in the following, the generalization to multiple oscillators is straightforward. The probability that a positive value of 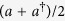 is obtained is given with the Wigner function as follows:


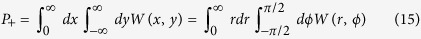


where we have used the polar coordinates. Using Eqs (13) and (14), we obtain


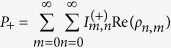


where


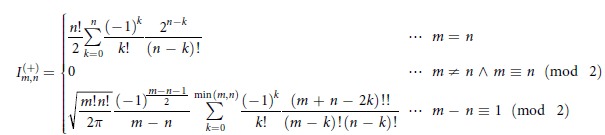


Similarly, the probability that a negative value of 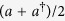 is obtained is given by


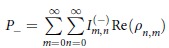


where


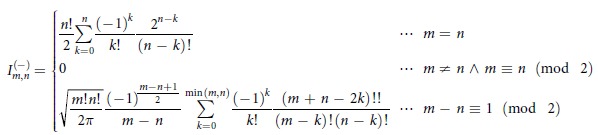


## Additional Information

**How to cite this article**: Goto, H. Bifurcation-based adiabatic quantum computation with a nonlinear oscillator network. *Sci. Rep.* **6**, 21686; doi: 10.1038/srep21686 (2016).

## Supplementary Material

Supplementary Information
