# Supplementary material for: Bifurcation-based adiabatic quantum computation with a nonlinear oscillator network
Source: Sci Rep. 2016 Feb 22;6:21686. doi: 10.1038/srep21686 (PMC4761947; doi:10.1038/srep21686)
Supplement: Supplementary Information [file srep21686-s1.pdf]

Supplementary Information

Bifurcation-based adiabatic quantum computation with a nonlinear oscillator network

Hayato Goto

Frontier Research Laboratory, Corporate Research & Development Center, Toshiba Corporation, 1, Komukai Toshiba-cho, Saiwai-ku, Kawasaki-shi, 212-8582, Japan.

1. Classical adiabatic evolution for a single KPO

Here we explain why the state of a single KPO changes along one of the stable branches in the classical model, as shown in Figs. 1a and 1b.

This result can be understood as follows. The system with constant p is conservative with the following conserved quantity:

$$E(x,y)=\frac{\Delta}{2}\left(x^2+y^2\right)+\frac{K}{4}\left(x^2+y^2\right)^2-\frac{p}{2}\left(x^2-y^2\right) \quad \text{(S1)}$$

Thus, the trajectories are given by contours of $E(x,y)$. Examples of the trajectories are shown in Figs. S1a ($p=0.9\Delta$) and S1b ($p=3\Delta$), where filled and open circles represent stable and unstable fixed points, respectively. Such figures are called phase portraits[1]. In the simulation shown in Figs. 1a and 1b, the trajectory is initially a small closed orbit around the origin. As p is increased slowly, the orbit changes while keeping its area $\int dxdy$ constant according to the adiabatic theorem in classical mechanics, where the area is called adiabatic invariance[18]. Thus, above the bifurcation point ($p>\Delta$), the orbit moves to one of the stable fixed points (local minima of the energy surface).

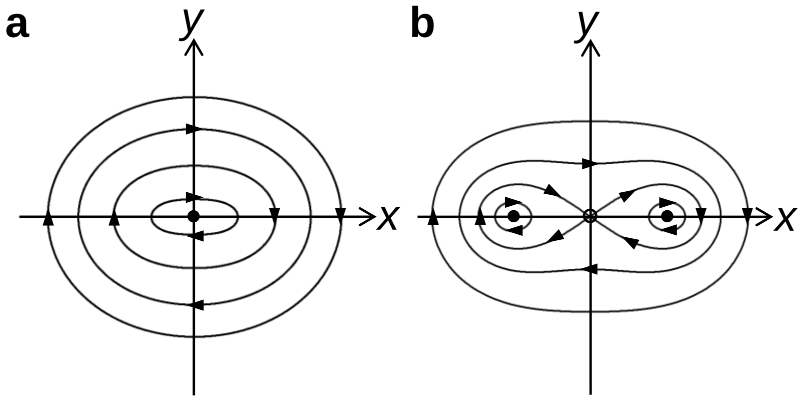

Figure S1 | Phase portrait for the classical model of a single KPO. Here Δ is set to K. **a**, **b**, Phase portraits for the classical model, where p is set as $p=0.9\Delta$ (**a**) and $p=3\Delta$ (**b**). In **a** and **b**, the filled and open circles represent the stable and unstable fixed points, respectively.

2. Proof of the condition for quantum adiabatic evolution

Here we prove that a sufficient condition for that $|0\rangle$ is the ground state of *H* with $p=0$ is that *M* defined by Eq. (6) becomes positive semidefinite.

The total Hamiltonian *H* with $p=0$ can be written as

$$H=\hbar\frac{K}{2}\sum_{i=1}^{N}a_i^{\dagger 2}a_i^2+\hbar\sum_{i=1}^{N}\sum_{j=1}^{N}M_{i,j}a_i^{\dagger}a_j \quad \text{(S2)}$$

Since $H|0\rangle=0$, it is sufficient to show that *H* is nonnegative.

Since *M* is a Hermitian matrix, *M* is diagonalized as $D=UMU^{\dagger}$, where *D* and *U* are a diagonal matrix and a unitary matrix, respectively.

Thus we obtain

$$H=\hbar\frac{K}{2}\sum_{i=1}^{N}a_i^{\dagger 2}a_i^2+\hbar\sum_{i=1}^{N}D_{i,i}b_i^{\dagger}b_i \quad \text{(S3)}$$

where $b_i=\sum_{J=1}^{N}U_{i,j}a_j$. The operator of this form is nonnegative when *K* and all $D_{i,i}$ are nonnegative. All $D_{i,i}$ are nonnegative by the assumption that *M* is positive semidefinite. Thus, the proof is completed.

This condition is satisfied by choosing Δ_i as Eq. (7). This is easily confirmed as follows. Consider the following quadratic form of real variables $\{\eta_i\}$:

$$\sum_{i=1}^{N}\sum_{j=1}^{N}M_{i,j}\eta_i\eta_j=\xi_0\sum_{i=1}^{N}\sum_{j=i+1}^{N}\left|J_{i,j}\right|\left(\eta_i-\frac{J_{i,j}}{\left|J_{i,j}\right|}\eta_j\right)^2$$

where Eq. (7) has been used. This quadratic form is always nonnegative. Therefore, *M* is positive semidefinite.

3. Simulation of the quantum computation for a two-spin Ising problem with a ferromagnetic coupling

As a simplest problem, we considered a two-spin problem with a ferromagnetic coupling: $J_{i,j}=J_{j,i}=1$. In this case, the answer is easy: $s_1=s_2=\pm 1$. From Eq. (9), we will obtain an entangled cat state:

$$|ECS(p)\rangle=\frac{\left|\sqrt{p/K}\right\rangle\left|\sqrt{p/K}\right\rangle+\left|-\sqrt{p/K}\right\rangle\left|-\sqrt{p/K}\right\rangle}{\sqrt{2\left(1+e^{-4p/K}\right)}} \quad \text{(S4)}$$

We numerically solved the Schrödinger equation with *H* in Eq. (5), where the Hilbert space was truncated at a "photon" number of 20 for each KPO, the initial state was set to $|0\rangle$, $\Delta_1=\Delta_2=\xi_0=0.5K$, and *p* is increased linearly from zero to 5*K*. Figure S2 shows the fidelity between the calculated state $|\psi(t)\rangle$ and the entangled cat state $|ECS(p(t))\rangle$ defined as $F=\left|\langle\psi|ECS\rangle\right|^2$. In Fig. S2, the upper and lower curves are the results for the computation times of 500/*K* and 200/*K*, respectively.

The high fidelities in Figs. S2 prove that the entangled cat state can be generated indeed. Thus, the present quantum computation provides a simple method for deterministic generation of such an intriguing quantum state via quantum adiabatic evolution. Figure S2 also shows that the more slowly *p* is increased, the higher the fidelity becomes. This is the feature of quantum adiabatic evolution.

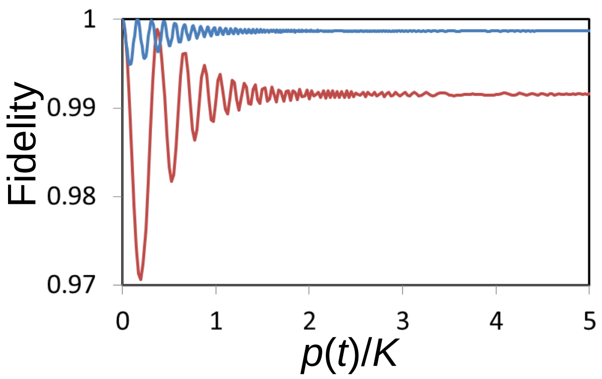

Figure S2 | Simulation result of the quantum computation for a two-spin Ising problem with a ferromagnetic coupling. The coupling coefficients are defined as $J_{1,2} = J_{2,1} = 1$. The fidelity is defined as $F = \left|\langle \psi | ECS \rangle\right|^2$, where $|\psi(t)\rangle$ is the calculated state and $|ECS(p(t))\rangle$ is the entangled cat state defined by Eq. (S4). The parameters are set as $\Delta_1 = \Delta_2 = \xi_0 = 0.5K$. p is increased linearly from zero to 5*K*. The upper and lower curves correspond to the computation times of 500/*K* and 200/*K*, respectively.

4. Approximate solution by the classical model

The equations of motion in the classical model for the present quantum computation are given by

$$\dot{x}_i = y_i\left[\Delta_i + p + K\left(x_i^2 + y_i^2\right)\right] - \xi_0 \sum_{j=1}^{N} J_{i,j} y_j$$

$$\dot{y}_i = x_i\left[-\Delta_i + p - K\left(x_i^2 + y_i^2\right)\right] + \xi_0 \sum_{j=1}^{N} J_{i,j} x_j$$

Here we show that the classical model can solve a relaxation problem of the Ising problem, where the relaxation is to replace the Ising spin s_i with a continuous variable ζ_i. We obtain an approximate solution for the original problem by identifying the sign of ζ_i as s_i.

First, we restate the Ising problem as the following energy is to be minimized:

$$E'_{\text{Ising}} = -\xi_0 \sum_{i=0}^{N}\sum_{j=0}^{N} J_{i,j} s_i s_j + \sum_{i=0}^{N} \Delta_i s_i^2 = \sum_{i=0}^{N}\sum_{j=0}^{N} M_{i,j} s_i s_j$$

Since $s_i^2 = 1$, this problem is equivalent to the original one.

The relaxation problem is defined as the following energy is to be minimized under the condition that $\sum_{i=1}^{N} \zeta_i^2 = N$:

$$E_c = \sum_{i=0}^{N}\sum_{j=0}^{N} M_{i,j} \zeta_i \zeta_j$$

where the constraint condition is necessary to find nontrivial solutions. Since *M* is positive semidefinite, the solution of the relaxation problem is given by the eigenvector of *M* for the smallest eigenvalue. Here it is also important that we can obtain a lower bound for the Ising energy from the solution of the relaxation problem.

On the other hand, the classical model can find such a vector at the first bifurcation point. Near the bifurcation point, both *x* and *y* are small. Ignoring the nonlinear terms, we obtain the following condition for the fixed points:

$$y_i(\Delta_i + p) - \xi_0 \sum_{j=1}^{N} J_{i,j} y_j = p y_i + \sum_{j=1}^{N} M_{i,j} y_j = 0$$

$$x_i(-\Delta_i + p) + \xi_0 \sum_{j=1}^{N} J_{i,j} x_j = p x_i - \sum_{j=1}^{N} M_{i,j} x_j = 0$$

These are characteristic equations for *M*. Since *M* is positive semidefinite and $p \geq 0$, $\{y_i\}$ cannot have nontrivial solutions. On the other hand, $\{x_i\}$ has a nontrivial solution at the bifurcation point, where *p* is the smallest eigenvalue of *M*. Then $\{x_i\}$ is the corresponding eigenvector.

Thus the classical model can find the solution for the relaxation problem of the Ising problem. This may be the reason why the classical model can find optimal solutions with high probability, as shown in Fig. 2c.
